# Plasma Matrix Metalloproteinases Signature as Biomarkers for Pediatric Tuberculosis Diagnosis: A Prospective Case–Control Study

**DOI:** 10.3390/diseases13060171

**Published:** 2025-05-27

**Authors:** Nathella Pavan Kumar, Syed Hissar, Arul Nancy, Kannan Thiruvengadam, Velayuthum V. Banurekha, Sarath Balaji, S. Elilarasi, N. S. Gomathi, J. Ganesh, M. A. Aravind, Dhanaraj Baskaran, Soumya Swaminathan, Subash Babu

**Affiliations:** 1 ICMR-National Institute for Research in Tuberculosis, Chennai 600031, India; kannan.t@nirt.res.in (K.T.); banurekha@nirt.res.in (V.V.B.); gomathisharma@nirt.res.in (N.S.G.); baskar.d@nirt.res.in (D.B.); doctorsoumya@yahoo.com (S.S.); 2 National Institutes of Health, National Institute for Research in Tuberculosis-International Center for Excellence in Research, Chennai 600031, India; arul.p@icerindia.org (A.N.); sbabu@icerindia.org (S.B.); 3 Institute of Child Health and Hospital for Children, Chennai 600008, India; sarath1731@gmail.com (S.B.); unga_elil@yahoo.com (S.E.); 4 Government Stanley Medical College and Hospital, Chennai 600001, India; stanleyganesh@gmail.com (J.G.); stanleyaravinda@gmail.com (M.A.A.); 5 M S Swaminathan Research Foundation, Chennai 600113, India; 6 Laboratory of Parasitic Diseases, National Institute of Allergy and Infectious Diseases, National Institute of Health, Bethesda, MD 20852, USA

**Keywords:** MMPs, biomarkers, tuberculosis

## Abstract

Diagnosing tuberculosis (TB) in children presents significant challenges, necessitating the identification of reliable biomarkers for accurate diagnosis. In this study, we investigated plasma matrix metalloproteinases (MMPs) and tissue inhibitors of metalloproteinases (TIMPs) as potential diagnostic markers. A prospective case–control study involved 167 children classified into confirmed TB, unconfirmed TB, and unlikely TB control groups. Plasma levels of MMPs (MMP 1, 2, 3, 7, 8, 9, 12, and 13) and TIMPs (TIMP 1, 2, 3, and 4) were measured using multiplex assays. Elevated baseline levels of MMP-1, MMP-2, MMP-7, MMP-9, TIMP-1, TIMP-2, TIMP-3, and TIMP-4 were observed in active TB cases compared to unlikely TB controls. Receiver operating characteristics (ROC) analysis identified MMP-1, MMP-2, MMP-9, and TIMP-1 as potential biomarkers with over 80% sensitivity and specificity. A three-MMP signature (MMP-1, MMP-2, and MMP-9) demonstrated 100% sensitivity and specificity. The findings suggest that a baseline MMP signature could serve as an accurate biomarker for diagnosing pediatric TB, enabling early intervention and effective management.

## 1. Introduction

Tuberculosis (TB) stands as a leading cause of global mortality, with pediatric TB significantly contributing to the overall burden, particularly in high-burden countries [1]. Pediatric TB poses a diagnostic conundrum owing to its paucibacillary nature, which renders current clinical and laboratory diagnostic tools less effective [2]. Compounding the issue is the challenge of obtaining sputum samples from young children, further complicating bacteriological confirmation. Hence, the demand for reliable, non-sputum-based point-of-care (POC) tests for pediatric TB diagnosis is urgent [3]. Host immune biomarkers in blood have emerged as an alternative for early TB detection and are prioritized in the WHO’s End TB strategy. Nevertheless, developing an effective POC diagnostic tool for pediatric TB remains daunting, given the unique characteristics of this population.

Matrix metalloproteinases (MMPs), pivotal in tissue oxidation and immunological defense, have surfaced as potential biomarkers. These proteolytic enzymes are essential in various physiological processes, including immune responses, inflammation, tissue repair, and matrix protein breakdown [4,5]. Concurrently, tissue inhibitors of metalloproteinases (TIMPs), integral to tissue remodeling and healing, counterbalance MMP-induced tissue damage [6]. Previous research in the pediatric population has indicated elevated levels of MMP1, 7, 8, and TIMP 1, 3 in children with pulmonary TB compared to those with extrapulmonary TB and healthy controls [7,8]. Similarly, studies in adults with pulmonary TB have shown increased circulating levels of MMP1, 2, 3, 7, 10, 12, and 13 compared to healthy controls [9]. Recent findings have also suggested that MMP and TIMP levels may serve as correlates of risk and prognostic biomarkers for treatment failure, relapse, and death in individuals with pulmonary TB, with MMPs being key mediators of TB pathology [10]. This study aims to assess the plasma concentrations of MMPs and TIMPs to distinguish between microbiologically confirmed, unconfirmed, and unlikely TB cases among prospectively recruited pediatric participants.

## 2. Methods

### 2.1. Study Population and Procedures

We conducted a prospective case–control study spanning the pediatric wards of the Institute of Child Health and Hospital for Children, Chennai, and Government Stanley Medical College and Hospital, Chennai, between February 2016 and March 2018. This study aimed to assess plasma matrix metalloproteinases (MMPs) and tissue inhibitors of metalloproteinases (TIMPs) as potential diagnostic markers for pediatric tuberculosis (TB).

A total of 195 children were screened during the recruitment phase, of which 167 were enrolled in the study (Table 1). This cohort comprised 44 children with microbiologically confirmed TB, 47 with unconfirmed TB, and 76 unlikely TB controls who presented with other respiratory ailments. To ascertain TB status, children underwent various diagnostic tests, including sputum samples or gastric aspirates for microbiological testing and Tuberculin Skin Test (TST) for immune response assessment.

For confirmed TB cases, microbiological positivity for tuberculosis was required, as evidenced by Xpert MTB/RIF, smear, or culture testing. Unconfirmed TB cases exhibited clinical features suggestive of TB, abnormal chest X-rays, or a history of household TB contact or positive response to anti-tuberculosis treatment (ATT), meeting at least two of the criteria. Unlikely TB controls were children with alternative diagnoses, such as COPD, viral pneumonia, bacterial pneumonia, or asthma/wheeze, who were either TST negative or TST positive with non-TB diagnoses [11].

At enrollment, all active TB cases (confirmed and unconfirmed) had no prior TB or ATT history, and positive TST results were defined as an induration of at least 10 mm in diameter at the site of tuberculin inoculation. Blood samples were collected from all participants using sodium heparin tubes and transported within 2 h to the Immunology lab for processing. Plasma samples were subsequently stored in a −80 °C freezer for future analysis. Notably, all confirmed and unconfirmed TB cases received ATT for 6 months.

The study also classified disease severity into two categories: severe TB, characterized by abnormal chest x-rays with AFB smear positivity alongside positive Xpert/MGIT/LJ results, and minimal TB, which included abnormal chest x-rays with negative AFB smear or normal chest x-rays with positive Xpert/MGIT/LJ results.

The detailed demographic and epidemiological data for the participants have been previously reported [12,13].

### 2.2. Metalloproteinase Assays

Plasma levels of matrix metalloproteinases (MMPs) and tissue inhibitors of metalloproteinases (TIMPs) were quantified using commercially available kits. The assay’s lowest detection limits ranged from 7.2 to 211.3 pg/mL for different MMPs and TIMPs. Circulating plasma levels were assessed using a commercially available Luminex Magpix Multiplex Assay system (Bio-Rad, Hercules, CA, USA). Additionally, MMP and TIMP levels were measured with commercially available kits: the Luminex Human Magnetic Assay 8-Plex and 4-Plex kits, both from R&D Systems.

### 2.3. Statistical Analysis

Geometric means were used for central tendency measurements. The Kruskal–Wallis test with Dunn’s multiple comparisons assessed significant differences between confirmed TB, unconfirmed TB, and unlikely TB. Receiver operator characteristics (ROC) curves evaluated the discriminatory power of each immune biomarker, with optimal biomarker combinations selected using CombiROC v.1.2. GraphPad PRISM Version 8.0 facilitated data analysis.

## 3. Results

### 3.1. Elevated Plasma Levels of MMPs and TIMPs in Pediatric TB

**Figure 1:**
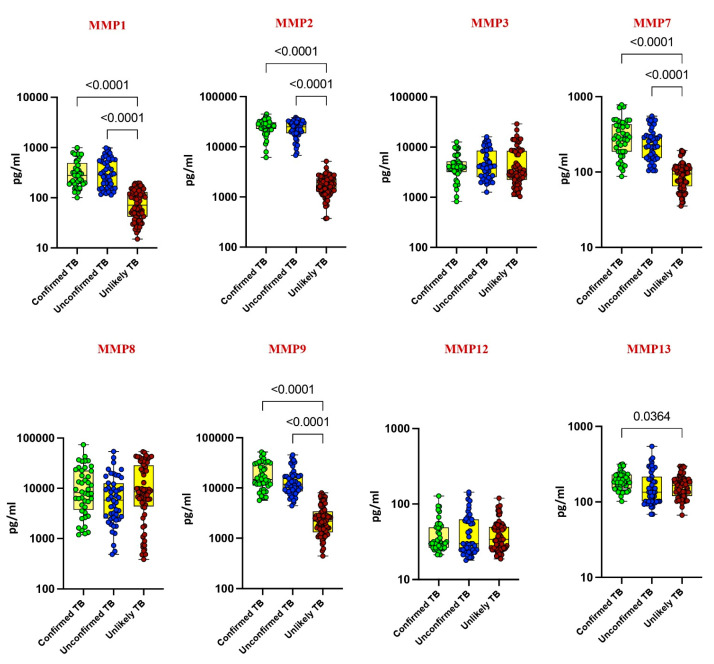
Elevated circulating levels of MMPs in children with active TB disease. The plasma levels of MMP 1, 2, 3, 7, 8, 9, 12, and 13 were measured in confirmed TB (n = 44), unconfirmed TB (n = 47), and unlikely TB (n = 76) individuals at baseline. The data are represented as scatter plots with each circle representing a single individual. *p* values were calculated using the Kruskal–Wallis test with Dunn’s post hoc for multiple comparisons.

**Figure 2:**
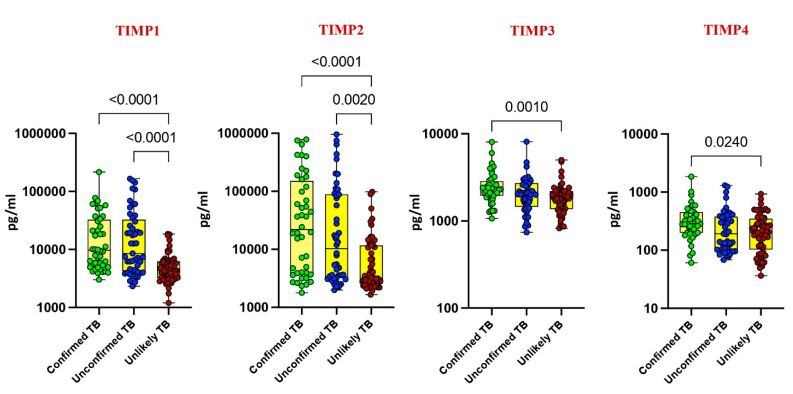
Elevated circulating levels of TIMPs in children with active TB disease. The plasma levels of TIMP 1, 2, 3, and 4 were measured in confirmed TB (n = 44), unconfirmed TB (n = 47), and unlikely TB (n = 76) individuals at baseline. The data are represented as scatter plots with each circle representing a single individual. *p* values were calculated using the Kruskal–Wallis test with Dunn’s post hoc for multiple comparisons.

Our investigation delved into the plasma levels of matrix metalloproteinases (MMPs) and tissue inhibitors of metalloproteinases (TIMPs) in pediatric tuberculosis (TB), comparing confirmed TB, unconfirmed TB, unlikely TB, and healthy controls. Significantly elevated levels of MMP-1, MMP-2, MMP-7, MMP-9, TIMP-1, and TIMP-2 were noted in confirmed and unconfirmed TB cases compared to unlikely TB and healthy controls (*p* < 0.001). For instance, MMP-1 exhibited a geometric mean (GM) of 295.8 pg/mL in confirmed TB, while it was 303 pg/mL in unconfirmed TB and 70 pg/mL in unlikely TB. Similarly, MMP-2 displayed a GM of 24,719 pg/mL in confirmed TB, 22,163 pg/mL in unconfirmed TB, and 1646 pg/mL in unlikely TB (Figure 1 and Figure 2).

Conversely, as shown in Figure 1 and Figure 2, MMP-13, TIMP-3, and TIMP-4 showed elevated levels exclusively in confirmed TB cases (*p* < 0.001). For instance, MMP-13 had a GM of 188 pg/mL in confirmed TB, while it was 150 pg/mL in unlikely TB. Likewise, TIMP-3 exhibited a GM of 2373 pg/mL in confirmed TB, contrasting with 1791 pg/mL in unlikely TB. No significant differences were observed for MMP-3, MMP-8, and MMP-12 among the study groups. These findings underscore the potential of MMPs and TIMPs as diagnostic markers for pediatric TB.

### 3.2. Plasma MMPs Associated with Disease Severity

**Figure 3:**
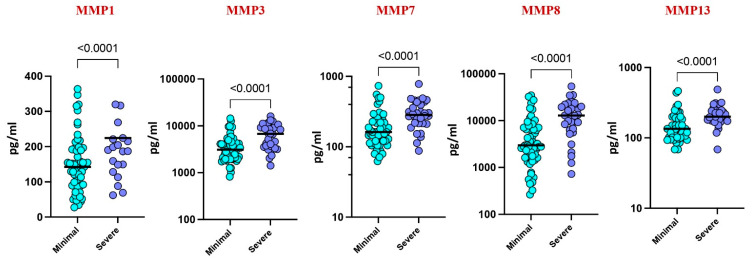
Plasma MMPs are associated with disease severity. The plasma levels of MMP 1, 3, 7, 8, and 13 were measured among the active TB children with minimal TB (n = 59) and severe TB (n = 32) individuals at baseline. The data are represented by scatter plots with each circle representing a single individual. *p* values were calculated using the Mann–Whitney U test.

Upon stratifying by disease severity, based on chest X-ray and smear reports, higher levels of MMP-1 (*p* = 0.0155), MMP-3 (*p* = 0.419), MMP-7 (*p* = 0.0038), MMP-8 (*p* = 0.0018), and MMP-13 (*p* = 0.0281) were observed in severe cases compared to those with minimal illness. These findings suggest a correlation between elevated MMP levels and disease severity in pediatric TB (Figure 3).

### 3.3. MMPs as Discriminatory Markers

**Figure 4:**
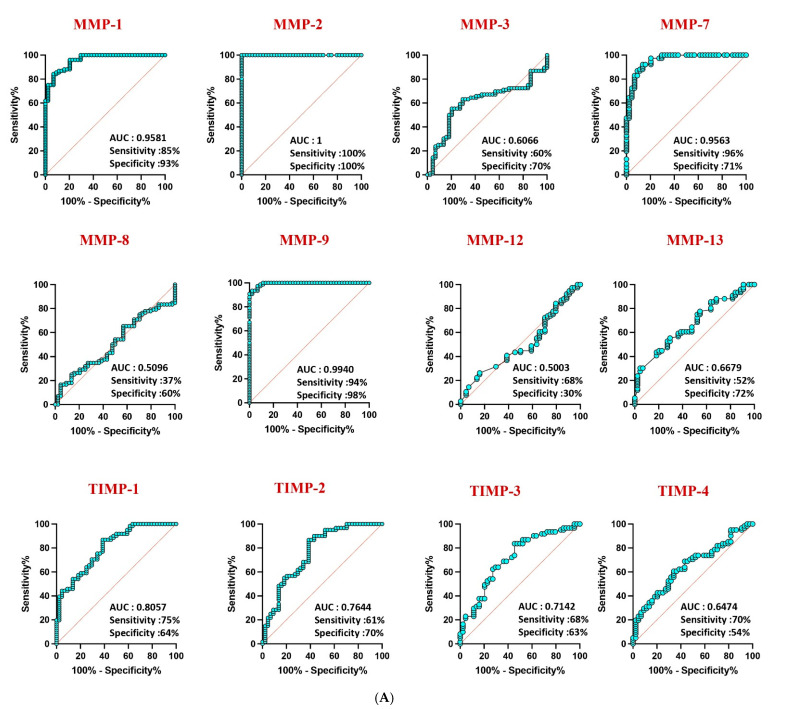

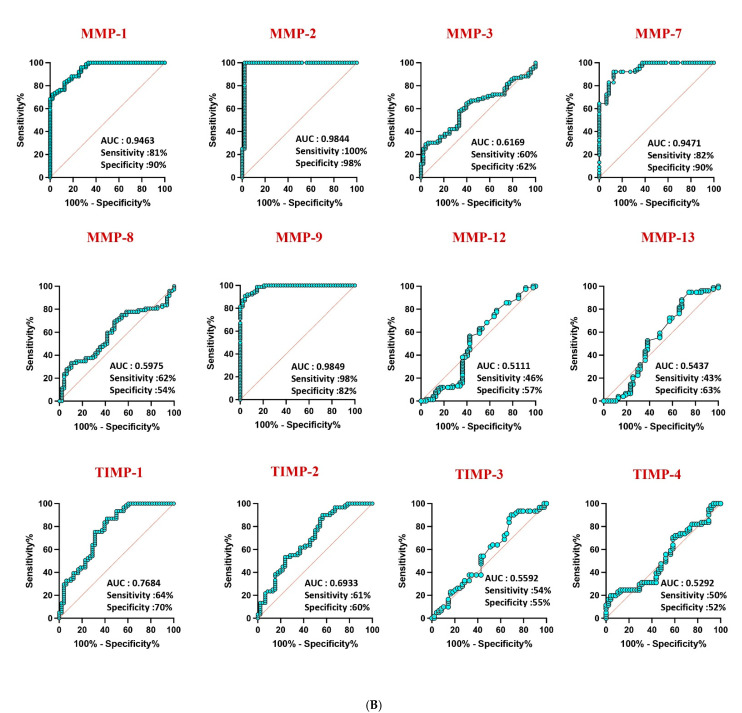
ROC analysis to estimate the discriminatory power of MMP in children with active TB disease and unlikely TB. ROC analysis to estimate the sensitivity, specificity and AUC was performed using MMP 1, 2, 3, 7, 8, 9, 12, and 13 and TIMP 1, 2, 3, and 4 to estimate the capacity of these factors to distinguish individuals with (**A**) confirmed TB vs. unlikely TB and (**B**) unconfirmed TB vs. unlikely TB. ROC = receiver operator characteristics.

Receiver operating characteristic (ROC) analysis highlighted the robust discriminatory power of MMP-1 (AUC = 0.9581), MMP-2 (AUC = 1), and MMP-9 (AUC = 0.9940) in distinguishing confirmed TB from unlikely TB, with high area under the curve (AUC) values (Figure 4A). Similar discriminatory potential was observed for unconfirmed TB vs. unlikely TB. However, other MMPs and TIMPs exhibited weaker sensitivity and specificity, underscoring the differential diagnostic capacity of specific MMPs in pediatric TB (Figure 4B).

### 3.4. Enhanced Discrimination with Biomarker Combinations

**Figure 5:**
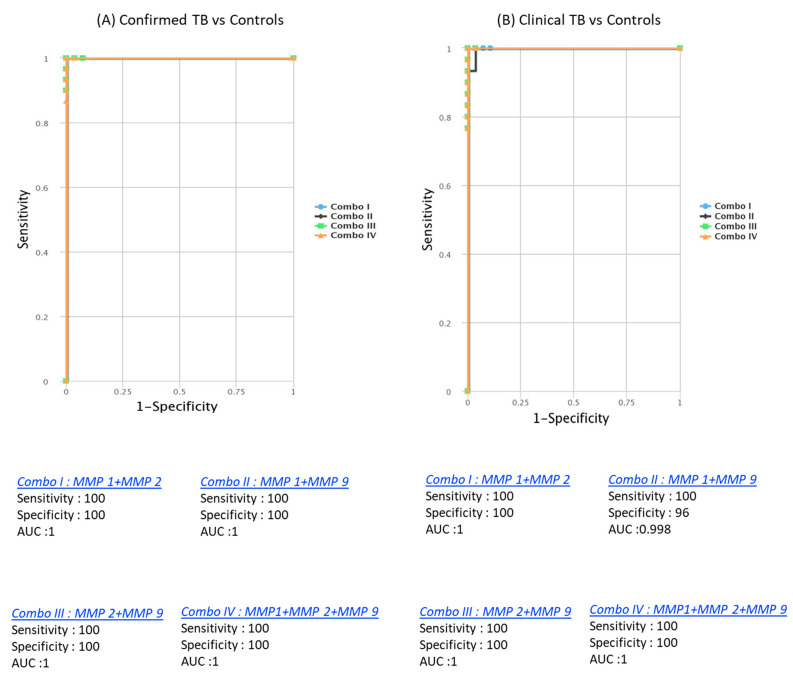
Identification of biomarkers showing the strongest association using a combination of MMP biomarkers in active TB disease. CombiROC model analysis shows the chemokines with the highest accuracy in discriminating confirmed TB and unconfirmed TB disease from unlikely TB. ROC curves for comparing multiple markers and their combinations between confirmed TB and unconfirmed TB versus unlikely TB. (**A**) Confirmed TB vs. unlikely TB; (**B**) unconfirmed TB vs. unlikely TB are shown.

CombiROC analysis unveiled the significant improvement in discriminatory power with combinations of MMPs. Dual combinations, such as MMP1/MMP2 (AUC = 1, sensitivity = 100%, specificity = 100%), MMP1/MMP9 (AUC = 0.986, sensitivity = 97%, specificity = 90%), MMP2/MMP9 (AUC = 1, sensitivity = 100%, specificity = 100%), and triple combinations like MMP1/MMP2/MMP9 (AUC = 1, sensitivity = 100%, specificity = 100%), demonstrated outstanding predictive performance. These combinations achieved high AUC, sensitivity, and specificity in discriminating both confirmed from unlikely TB cases. Similarly, for discriminating both unconfirmed from unlikely TB cases, dual combinations like MMP1/MMP2 (AUC = 1, sensitivity = 100%, specificity = 100%), MMP1/MMP9 (AUC = 0.973, sensitivity = 94%, specificity = 90%), MMP2/MMP9 (AUC = 0.992, sensitivity = 97%, specificity = 100%), and triple combinations such as MMP1/MMP2/MMP9 (AUC = 1, sensitivity = 100%, specificity = 100%) were highly effective (Figure 5). These findings underscore the utility of a signature comprising two or three MMPs as accurate biomarkers for discriminating active TB disease in pediatric populations from controls, offering enhanced sensitivity and specificity.

## 4. Discussion

Children and adolescents constitute a clinically significant demographic with heightened susceptibility to tuberculosis (TB) [14]. Pediatric TB manifests with a rapid progression from Mycobacterium tuberculosis infection to active disease, influenced by various factors including age, nutritional status, immune competence, genetic predisposition, and the severity of the initial infection. Despite contributing significantly to the overall TB burden, diagnosing TB in children poses considerable challenges due to low positivity rates in conventional diagnostic methods [15,16].

Current diagnostic approaches for pediatric TB rely on clinical symptoms, radiological findings, and the detection of M. tuberculosis in respiratory samples, gastric aspirates, or sputum [17]. However, these methods often fall short, particularly in resource-limited settings, highlighting the critical need for effective non-sputum-based point-of-care (POC) tests. Moreover, the swift progression from infection to disease in children underscores the urgency for early and accurate diagnostic modalities.

Previously, in 2013, our research identified elevated levels of MMP-1, MMP-7, MMP-8, TIMP-1, and TIMP-3 in pulmonary or extrapulmonary TB compared to controls [8]. This suggests that higher MMP levels in TB-infected children could potentially serve as biomarkers to distinguish individuals with TB disease from healthy controls. However, our earlier study had limitations, including a small sample size and the absence of children with latent TB or other pulmonary infections. In the current study, well-defined groups within the study cohort address these limitations.

Additionally, our research has explored combinations of MMP-7, C-reactive protein (CRP), and lipopolysaccharide-binding protein (LBP) as markers with high accuracy in discriminating between children with active TB and healthy controls [7]. Furthermore, recent findings from our group have highlighted baseline cytokine signatures (TNFα, IL-2, IL-17A) and chemokine signatures (CCL1/CXCL1/CXCL10) as potential accurate biomarkers for pediatric TB diagnosis [12,13]. These studies collectively underscore the ongoing efforts to identify reliable biomarkers and diagnostic tools to improve TB detection and management in pediatric populations.

The primary objective of this study was to assess the diagnostic utility of emerging immune biomarkers, specifically matrix metalloproteinases (MMPs), in pediatric tuberculosis (TB). Focusing on children with microbiologically confirmed TB, unconfirmed TB, and those unlikely to have TB, the study unveiled significantly elevated levels of MMPs (1, 2, 7, 9) and tissue inhibitors of metalloproteinases (TIMPs) in both confirmed and unconfirmed TB cases compared to controls. These findings suggest the promising potential of MMPs as blood-based biomarkers for distinguishing TB cases from those without the disease. Furthermore, the study demonstrated that combining different MMPs enhanced the discriminatory power, offering high sensitivity and specificity in distinguishing TB cases from controls. Particularly, the multi-biomarker panel comprising MMP-1/MMP-2, MMP-1/MMP-9, MMP-2/MMP-9, and MMP-1/MMP-2/MMP-9 showed robust results, exhibiting the highest values for sensitivity and specificity.

While some MMPs and TIMPs have been previously investigated in the immunopathology of pediatric TB, a comprehensive analysis of a wide panel of MMPs and TIMPs in confirmed TB, unconfirmed TB, and unlikely TB cases has not been undertaken until now. This study introduces a novel three-immune biosignature of MMPs capable of efficiently discriminating confirmed and unconfirmed TB from unlikely TB with good accuracy. Additionally, our findings suggest that regardless of diagnosis, host immune responses remain unaltered, indicating a consistent pattern across TB cases.

Expanding on our earlier report in 2013, which highlighted the elevation of MMP-1, MMP-7, MMP-8, TIMP-1, and TIMP-3 in pulmonary or extrapulmonary TB, this current study addresses previous limitations by incorporating a well-defined study cohort, including children with latent TB and other pulmonary infections [8]. Notably, combinations of MMP-7, C-reactive protein (CRP), and lipopolysaccharide-binding protein (LBP) emerged as markers with high accuracy in differentiating between children with active TB and healthy controls [7]. This underscores the pivotal role of MMPs as potent immune biomarkers for diagnosing pediatric TB.

In studies involving adult pulmonary tuberculosis (TB) patients, increased levels of matrix metalloproteinases (MMPs), including MMP-1, 2, 3, 7, 8, and 9, have been observed in various biological fluids such as sputum, pleural fluid, and bronchoalveolar lavage (BAL) fluids [18,19]. Additional investigations have reported elevated systemic levels of MMP-1 and MMP-19 in TB patients compared to controls [20,21]. Additionally, several published studies in adult populations have reported elevated MMP-9 levels in various respiratory infections, such as community-acquired pneumonia. In our cohort, to ensure that the responses are specific to TB disease, we used a comparative group of children with unlikely TB. These children visited the respiratory clinic for seasonal respiratory illnesses, helping to distinguish TB-specific responses from those associated with other respiratory conditions [22] (Yang et al., 2005). Furthermore, the severity of TB disease has been correlated with MMP levels, with comorbid conditions such as diabetes mellitus exacerbating clinical severity [9]. Recent findings from our group have further solidified the role of MMPs and tissue inhibitors of metalloproteinases (TIMPs) as correlates of risk and prognostic biomarkers for treatment failure, relapse, and death in individuals with pulmonary TB [10]. These data align with existing evidence, underscoring the association of elevated MMPs with disease severity and bacterial burden in TB. Collectively, MMPs emerge as pivotal contributors linked to the pathology of TB, making them promising candidates for biomarkers in pediatric TB diagnosis. Limitations of our study include a limited sample size, the absence of a validation cohort in a different geographical location, and the lack of inclusion of healthy control children. Additionally, the cost-effectiveness of this assay was also not addressed. Addressing these limitations in future research will be important for strengthening the validity and applicability of our conclusions.

The strengths of this study lie in the well-characterized participant groups and the inclusion of individuals deemed unlikely to have TB, enabling a robust comparison of MMP and TIMP responses among confirmed, unconfirmed, and improbable TB cases. While the findings are encouraging, further research in diverse endemic communities is warranted to validate MMPs as potential diagnostic tools for pediatric TB.

## 5. Conclusions

This study offers valuable insights into the potential of MMPs as immune biomarkers for pediatric TB diagnosis. The identification of reliable biomarkers holds promise for the development of effective, non-invasive diagnostic tools, addressing the unique challenges posed by TB in children.

## Figures and Tables

**Table 1:** Study demographics.

Demographic Characteristic	Confirmed TB	Unconfirmed TB	Unlikely TB
Number of subjects recruited	44	47	76
Gender (Male/Female)	16/28	28/19	36/40
Median Age (Range) (in years)	7 (1–13)	8 (1–12)	6 (1–14)
Bacterial Burden:	11/33/0	0/0/47	-
High burden/Low burden/No burden			
Tuberculin Skin Test: (Positive/Negative)	32/16	35/12	45/31

## Data Availability

All data generated or analyzed during this study are included in this published article.
